# Infection associated with CDK4/6 inhibitors: a pharmacovigilance analysis of the FDA adverse event reporting system database

**DOI:** 10.3389/fphar.2024.1371346

**Published:** 2024-07-01

**Authors:** Jinhua Chen, Linlin Tang, Wenping Song, Cuicui Sun, Wenzhou Zhang

**Affiliations:** ^1^ Department of Pharmacy, The Affiliated Cancer Hospital of Zhengzhou University and Henan Cancer Hospital, Henan Engineering Research Center for Tumor Precision Medicine and Comprehensive Evaluation, Henan Provincial Key Laboratory of Anticancer Drug Research, Zhengzhou, China; ^2^ Department of Pharmacy, Affiliated Hospital of Weifang Medical University, Weifang, China; ^3^ Department of Pharmacy, Qilu Hospital of Shandong University, Ji’nan, China

**Keywords:** CDK4/6 inhibitor, FAERS, infection, adverse events, ribociclib

## Abstract

**Introduction:**

Cyclin-dependent kinase 4 and 6 (CDK4/6) inhibitors are first-line treatments for hormone receptor-positive/human epidermal growth factor receptor 2-negative breast cancer. With their increasing clinical use, infection-related adverse events (AEs) associated with CDK4/6 inhibitors have been widely reported in recent years. This study aimed to analyze the occurrence of infections associated with the CDK4/6 inhibitors (palbociclib, ribociclib and abemaciclib) based on the real-world data from the US Food and Drug Administration Adverse Event Reporting System (FAERS) database.

**Methods:**

Data were extracted from the FAERS database between 2015Q1 and 2022Q3. The clinical characteristics of patients with primary suspected infection-related AEs were analyzed. A disproportionality analysis was performed to investigate the potential association between AEs and CDK4/6 inhibitors. The influencing factors were evaluated using Pearson’s chi-square test.

**Results:**

Reports of infection-related AEs associated with ribociclib accounted for 8.58% of the total reports of AEs associated with ribociclib, followed by palbociclib (2.72%) and abemaciclib (1.24%). Ribociclib (67.65%) was associated with more serious outcome events than palbociclib (30%) or abemaciclib (48.08%). The sex and age were not associated with outcome severity. Disproportionality analysis showed that fourteen, sixteen and two infection-related preferred terms were detected for palbociclib, ribociclib and abemaciclib, respectively.

**Conclusion:**

Infection-related AEs were highly associated with three CDK4/6 inhibitors, especially palbociclib and ribociclib, based on the real-world data from the FAERS database. However, further causality assessment is required.

## 1 Introduction

Breast cancer is the most frequently diagnosed cancer and the second leading cause of cancer-associated deaths in women worldwide (Giaquinto et al., 2022). Approximately 70% of breast cancer patients are positive for hormone receptors (estrogen receptor and/or progesterone receptor) and lack human epidermal growth factor receptor 2 (HER2) overexpression (Dai et al., 2015). Cyclin-dependent kinase 4 and 6 (CDK4/6) inhibitors are an important class of medications for targeted therapy for hormone receptor (HR)-positive/HER2-negative breast cancer (Morrison et al., 2023). Three CDK4/6 inhibitors, palbociclib, ribociclib, and abemaciclib, have been widely used as first-line treatments for patients with HR-positive/HER2-negative breast cancer (Guo et al., 2023; Elfgen and Bjelic-Radisic, 2021). Although they exhibit favorable outcomes, adverse reactions, such as neutropenia, gastrointestinal toxicity, diarrhea and pulmonary embolism, pose a great challenge to their clinical application (Thill and Schmidt, 2018). Additionally, it is noteworthy that some differences in adverse reactions to these three CDK4/6 inhibitors have been reported. In particular, neutropenia is the most common adverse reaction to palbociclib and ribociclib, and gastrointestinal toxicity is strongly associated with abemaciclib (Sammons et al., 2017).

Infectious diseases are responsible for numerous deaths and financial burdens worldwide (Omosigho et al., 2023; Fan et al., 2018). Neutropenia caused by anti-tumor therapy is a high-risk factor for infection (Joudeh et al., 2023; Villeneuve and Aftandilian, 2022). These CDK4/6 inhibitors cause reversible bone marrow suppression by inducing cell cycle arrest at the G1-S phase, which is different from the irreversible bone marrow suppression caused by cytotoxic chemotherapy (Bas et al., 2022; Hu et al., 2016). Therefore, bone marrow function can recover after the withdrawal of CDK4/6 inhibitors. According to the manufacturer’s instructions, infection is a common adverse reaction associated with CDK4/6 inhibitors. Recent studies have reported CDK4/6 inhibitor-related infections in post-marketing setting (Algwaiz et al., 2021; Rajendran et al., 2021; Sarkisian et al., 2020; Okayasu et al., 2023; Felip et al., 2020). Given the potential clinical benefits, a comprehensive study of infection-related adverse events (AEs) of these three CDK4/6 inhibitors in post-marketing setting is needed to better evaluate the occurrence of infection.

The USA Food and Drug Administration (FDA) Adverse Event Reporting System (FAERS) database, the largest pharmacovigilance database, is a public spontaneous reporting system (Rodriguez et al., 2001). Several case reports on AEs associated with FDA-approved medications available in the FAERS database (Sakaeda et al., 2013). It has been widely used to identify novel signals of medications and alert physicians and patients to pay attention to potential medication-related AEs (Javed and Kumar, 2024; Sharma et al., 2023; Jain et al., 2023; Sharma and Kumar, 2022). Therefore, this study is of great significance for evaluating the occurrence of infection-related AEs for CDK4/6 inhibitors based on real-world data from the FAERS database from 2015Q1 to 2022Q3. A disproportionality analysis was conducted to assess the potential association between infection-related AEs and CDK4/6 inhibitors. This study aimed to provide a comprehensive understanding of the occurrence of CDK4/6 inhibitor-associated infections.

## 2 Materials and methods

### 2.1 Data sources

This study was a pharmacovigilance analysis of infection-related AEs associated with CDK4/6 inhibitors based on the FAERS database. Data from the FAERS database were retrieved using OpenVigil 2.1. OpenVigil 2.1, a web-based pharmacovigilance analysis tool, provides physicians and pharmacists with an intuitive access to the FAERS data using the openFDA online interface of theFDA (Böhm et al., 2016). Three CDK4/6 inhibitors, namely, palbociclib, ribociclib and abemaciclib, were used to obtain reporting data from the FAERS database. The marketing approval times for palbociclib, ribociclib and abemaciclib by the FDA were February 2015, March 2017 and September 2017, respectively; therefore, we extracted all AE reports from 2015Q1 to 2022Q3 from the FAERS database. The systemic organ classes (SOCs) and preferred terms (PTs) were coded based on the Medical Dictionary for Regulatory Activities (MedDRA, version 25.1).

### 2.2 Signal mining

Disproportionality analysis was used to evaluate the potential association between AEs and CDK4/6 inhibitors by calculating the reporting odds ratio (ROR) and 95% confidence interval (CI) (Table 1). Infection-related PTs were considered highly associated with the treatment of CDK4/6 inhibitors when the case numbers were ≥3 and the lower limit of the two-sided 95% CI was >1. PTs that met these criteria were screened for further analysis.

**TABLE 1:** The algorithm used for signal detection.

Algorithm	Equation	Criteria
ROR	ROR=(a/c)/(b/d)	a≥3, 95%CI ≥ 1
	95%CI = e^ln(ROR)±1.96(1/a+1/b+1/c+1/d)^0.5^	

Equation: a, number of reports containing both the target drug and target adverse drug reaction; b, number of reports containing other adverse drug reaction of the target drug; c, number of reports containing the target adverse drug reaction of other drugs; d, number of reports containing other drugs and other adverse drug reactions. 95%CI, 95% confidence interval; ROR, reporting odds ratio.

### 2.3 Data processing procedure

**FIGURE 1:**
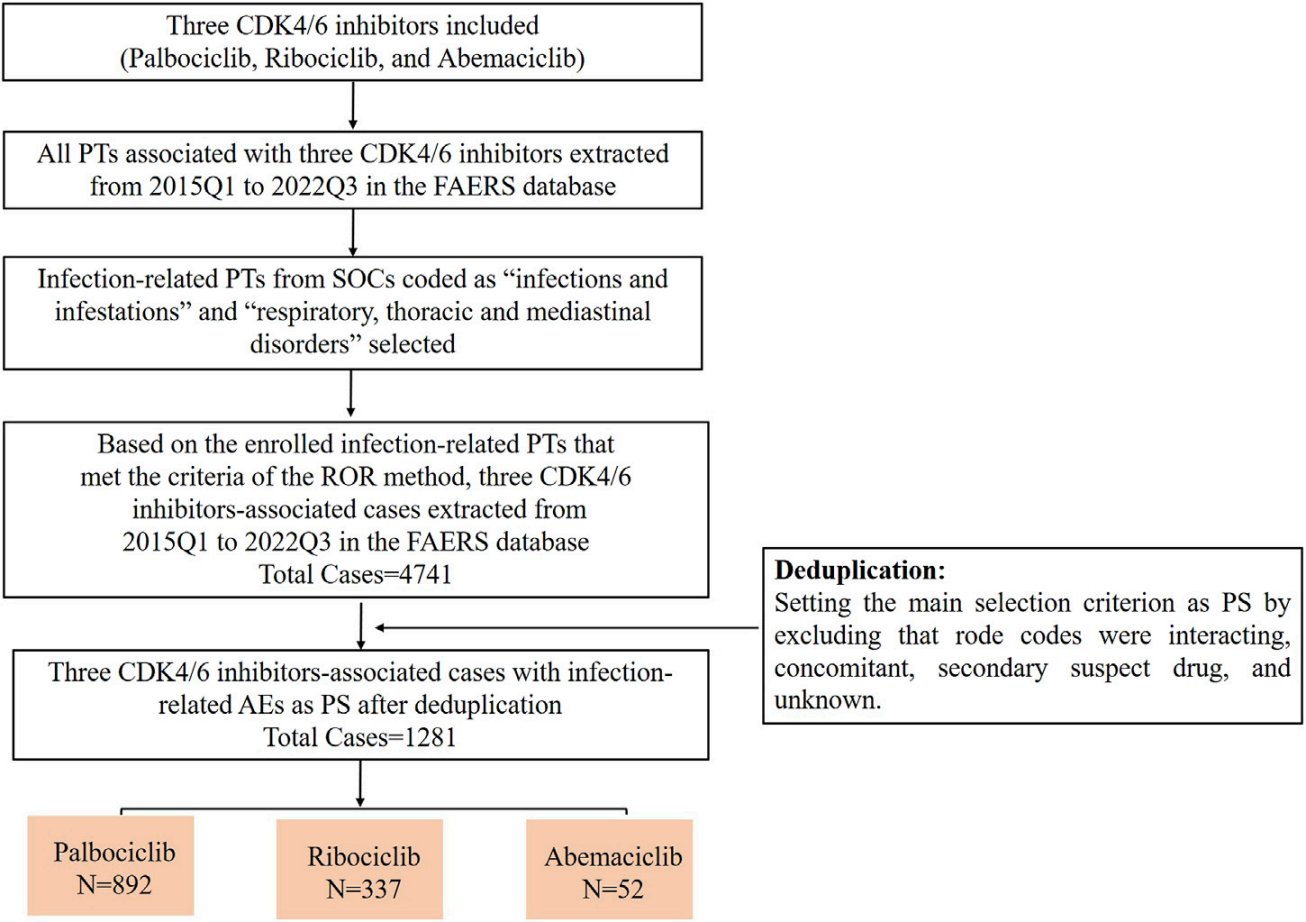
The flow chart of screening infection-related AEs for these three CDK4/6 inhibitors from 2015Q1 to 2022Q3 in the FAERS database.

First, we extracted all PTs associated with each CDK4/6 inhibitor between 2015Q1 and 2022Q3 from the FAERS database. Next, the extracted PTs were grouped into different SOCs. Thirdly, infection-related PTs from the SOCs coded as “infections and infestations” and “respiratory, thoracic and mediastinal disorders” were selected, and analyzed using the ROR method. Infection-related PTs that met the criteria of the ROR method were screened for further analysis. Fourth, all case data were retrieved from 2015Q1 to 2022Q3 using the enrolled infection-related PTs for each CDK4/6 inhibitor. Lastly, cases of primary suspect (PS) were selected and used for subsequent analysis by excluding those in which role codes were interacting, concomitant, secondary suspect, or unknown (Figure 1).

Descriptive analyses of the clinical characteristics of the enrolled cases were performed, including sex, age, reporting region, reporting year, and outcomes. Serious outcomes included hospitalization initial or prolonged, life-threatening, disability, and death.

### 2.4 Statistical analysis

Descriptive analyses were performed to evaluate clinical characteristics. The influencing factors were compared using Pearson’s chi-square test. All data mining and statistical analyses were performed using Microsoft Excel 2019, SPSS, and GraphPad Prism 5.

## 3 Results

### 3.1 Infection-related PTs for each CDK4/6 inhibitor from 2015Q1 to 2022Q3 in the FAERS

**FIGURE 2:**
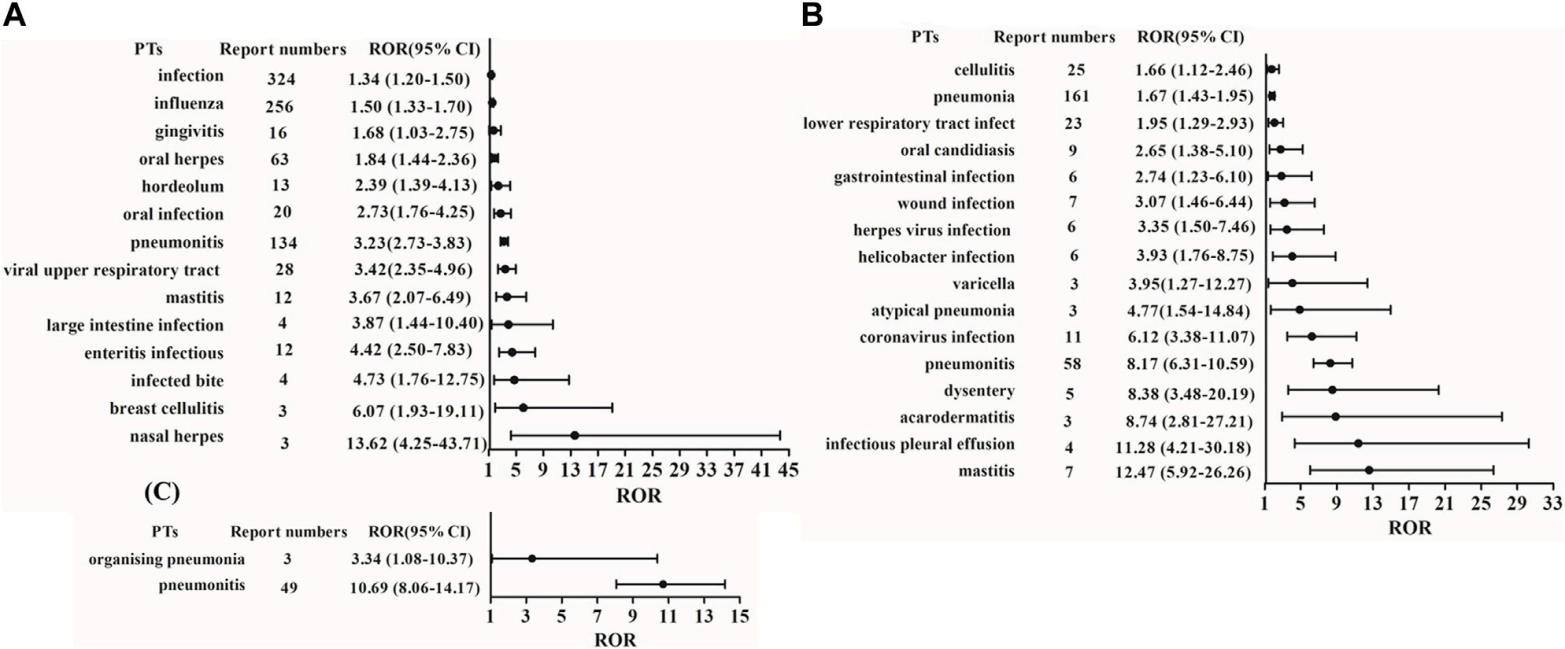
ROR and 95% CI values of infection-related PTs for these three CDK4/6 inhibitors. **(A)** Palbociclib; **(B)** Ribociclib; **(C)** Abemaciclib.

In this study, we first extracted all PTs for each CDK4/6 inhibitor from 2015Q1 to 2022Q3, and screened infection-related PTs from two SOCs coded as “infections and infestations” and “respiratory, thoracic and mediastinal disorders” (Supplementary Table S1). Based on disproportionality analysis by calculating the ROR value of PTs, fourteen infection-related PTs were associated with palbociclib treatment, including infection, influenza, gingivitis, oral herpes, hordeolum, oral infection, pneumonitis, viral upper respiratory tract infection, mastitis, large intestine infection, enteritis infectious, infected bite, breast cellulitis, and nasal herpes. Nasal herpes had the lowest number of cases among the fourteen PTs but the highest ROR value (Figure 2A). Sixteen infection-related PTs were associated with ribociclib treatment, including cellulitis, pneumonia, lower respiratory tract infection, oral candidiasis, gastrointestinal infection, wound infection, herpes virus infection, *helicobacter* infection, varicella, atypical pneumonia, coronavirus infection, pneumonitis, dysentery, acarodermatitis, infectious pleural effusion, and mastitis (Figure 2B). In addition, two infection-related PTs (organising pneumonia and pneumonitis) were strongly associated with abemaciclib treatment (Figure 2C).

### 3.2 Infection-related AEs among CDK4/6 inhibitor users from 2015Q1 to 2022Q3 in the FAERS

We further analyzed the occurrence of infection-related AEs in patients treated with the three CDK4/6 inhibitors. Based on the PTs enrolled for each CDK4/6 inhibitor, we extracted infection-related AEs from 2015Q1 to 2022Q3 from the FAERS. As shown in Table 2, the infection-related AEs for palbociclib accounted for 2.72% (2945/108458) of the total palbociclib-associated AEs, ribociclib accounted for 8.58% (1681/19586), and abemaciclib accounted for 1.24% (115/9266). To mitigate the effects of non-CDK4/6 inhibitor factors to some extent, we selected infection-related AEs as PS by excluding cases in which infection-related AEs may have occurred due to interacting drugs, concomitant drugs, secondary suspected drugs, and other unknown (Figure 1). The results showed that the proportions of infection-related AEs as PS for palbociclib, ribociclib and abemaciclib were 30.29% (892/2945), 20.05% (337/1681), and 45.22% (52/115), respectively.

**TABLE 2:** Distribution of AEs associated with these three CDK4/6 inhibitors.

Drug names	All AEs for each CDK4/6 inhibitor, n	Infection-related AEs for each CDK4/6 inhibitor, n (%)	Infection-related AEs for each CDK4/6 inhibitor as PS, n (%)
Palbociclib	108458	2945 (2.72)	892 (30.29)
Ribociclib	19586	1681 (8.58)	337 (20.05)
Abemaciclib	9266	115 (1.24)	52 (45.22)

**FIGURE 3:**
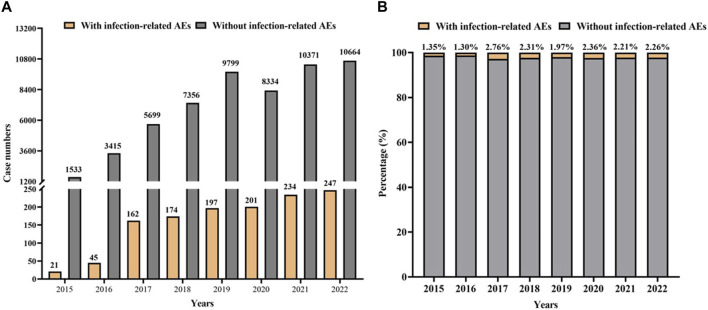
Statistic on the occurrence of infection-related AEs for CDK4/6 inhibitors from the FAERS during 2015Q1-2022Q3. **(A)** Case numbers of CDK4/6 inhibitors with infection-related AEs as PS *versus* without infection-related AEs as PS. **(B)** The proportion of reports with infection-related AEs as PS or without infection-related AEs in all reports for CDK4/6 inhibitors as PS.

Additionally, the number of cases with infection-related AEs as PS increased slightly but remained a relatively constant proportion (1.97%–2.76%) of the overall reports of AEs as PS in the years 2017–2022. The numbers and proportions were low in 2015 and 2016 because only palbociclib was approved at that time (Figure 3).

### 3.3 Descriptive analyses of cases with infection-related AEs

After identifying the cases of infection-related AEs as PS, we analyzed their clinical characteristics. As shown in Table 3, there were 1281 cases. Among them, there were 1206 (94.15%) cases for females, 17 (1.33%) for males, and 58 (4.53%) individuals with missing information about their sex. The median patient age was 64.75 years (interquartile range 30–94). The patients were divided into three age groups: <40 years, 40–64 years, and ≥65 years. They accounted for 2.73% (35/1281), 32.32% (414/1281), and 42.54% (545/1281), respectively, of the total. The majority of cases were from the United States (67.37%, 863/1281), followed by Germany (5.00%, 64/1281) and the United Kingdom (4.06%, 52/1281).

**TABLE 3:** Clinical characteristics of patients.

	Palbociclib (n, %)	Ribociclib (n, %)	Abemaciclib (n, %)	Total (n, %)
Sex				
Female	838 (93.95)	324 (96.14)	44 (84.62)	1206 (94.15)
Male	10 (1.12)	7 (2.08)	─	17 (1.33)
Unknown	44 (4.93)	6 (1.78)	8 (15.38)	58 (4.53)
Age (year)				
Median (interquartile)	65.01 (30–93)	63.78 (30–94)	64.83 (37–89)	64.75 (30–94)
<40	24 (2.69)	10 (2.97)	1 (1.92)	35 (2.73)
40–64	323 (36.21)	83 (24.63)	8 (15.38)	414 (32.32)
≥65	415 (46.52)	115 (34.12)	15 (28.85)	545 (42.54)
Unknown	130 (14.57)	129 (38.28)	28 (53.85)	287 (22.40)
Reporting years				
2015	21 (2.35)	─	─	21 (1.64)
2016	45 (5.04)	─	─	45 (3.51)
2017	152 (17.04)	10 (2.97)	─	162 (12.65)
2018	134 (15.02)	40 (11.87)	─	174 (13.58)
2019	130 (14.57)	58 (17.21)	9 (17.31)	197 (15.38)
2020	122 (13.68)	67 (19.88)	12 (23.08)	201 (15.69)
2021	120 (13.45)	93 (27.60)	21 (40.38)	234 (18.27)
2022	168 (18.83)	69 (20.47)	10 (19.23)	247 (19.28)
Reporting country				
United States	768 (86.10)	60 (17.80)	35 (67.31)	863 (67.37)
Germany	5 (0.56)	56 (16.62)	3 (5.77)	64 (5.00)
United Kingdom	9 (1.01)	40 (11.87)	3 (5.77)	52 (4.06)
Argentina	32 (3.59)	8 (2.37)	─	40 (3.12)
Canada	7 (0.78)	12 (3.56)	─	19 (1.48)
Puerto Rico	12 (1.35)	─	─	12 (0.94)
Japan	9 (1.01)	─	3 (5.77)	12 (0.94)
Other countries or unknown	50 (5.61)	161 (47.77)	8 (15.38)	219 (17.10)

### 3.4 Comparison between the severe and non-severe groups

Most cases of infection-related AEs as PS (40.36%, 517/1281) had serious outcome events, including hospitalization initial or prolonged, death, life-threatening, and disability. The proportions of serious outcome events for palbociclib, ribociclib, and abemaciclib were 29.60% (264/892), 66.77% (225/337), and 48.08% (25/52), respectively. Non-serious outcome events accounted for 24.20% (310/1281) of the total. The proportions of non-serious outcome events for palbociclib, ribociclib, and abemaciclib were 22.87% (204/892), 27.30% (92/337), and 26.92% (12/52), respectively (Table 4). Furthermore, we explored the influencing factors by comparing serious and non-serious groups. There was no statistically significant difference in sex (χ2 = 1.533, *p* = 0.2156) and age (χ2 = 3.775, *p* = 0.1515) (Table 5).

**TABLE 4:** Outcome events.

	Palbociclib (n, %)	Ribociclib (n, %)	Abemaciclib (n, %)	Total (n, %)
Hospitalization initial or prolonged	210 (23.54)	152 (45.10)	9 (17.31)	371 (28.96)
Death	50 (5.61)	62 (18.40)	9 (17.31)	121 (9.45)
Life-threatening	4 (0.45)	11 (3.26)	6 (11.54)	21 (1.64)
Disability	─	3 (0.89)	1 (1.92)	4 (0.31)
Others	204 (22.87)	92 (27.30)	14 (26.92)	310 (24.20)
Unkown	424 (47.53)	17 (5.04)	13 (25.00)	454 (35.44)

**TABLE 5:** Differences in clinical characteristics between severe and non-severe groups.

	Serious cases (n = 517)	Non-serious cases (n = 310)	χ2	*P*
Sex				
Female	500 (96.71)	293 (94.52)	1.533	0.2156
Male	6 (1.16)	1 (0.32)		
Unknown	11 (2.13)	17 (5.48)		
Age (year)				
<40	18 (3.48)	5 (1.61)	3.775	0.1515
40–64	171 (33.08)	96 (30.97)		
≥65	205 (39.65)	138 (44.52)		
Unknown	123 (23.79)	71 (22.90)		

## 4 Discussion

Although AEs associated with CDK4/6 inhibitors have been widely reported, comprehensive studies on infection-related AEs associated with CDK4/6 inhibitors are lacking (Thill and Schmidt, 2018). To the best of our knowledge, this study is the first pharmacovigilance analysis about infection-related AEs associated with CDK4/6 inhibitors using real-world case reports from the FAERS database in a post-marketing setting. In the present study, the occurrence of infection-related AEs associated with these three CDK4/6 inhibitors, palbociclib, ribociclib and abemaciclib, was discussed. Our study showed that the proportion of infection-related AEs for these three CDK4/6 inhibitors ranged from 1.24% to 8.58% during 2015Q1-2022Q3, with a higher prevalence in patients aged >40 year old.

Palbociclib was first approved in the United States in 2015 and was used in combination with letrozole to treat postmenopausal women with estrogen receptor (ER)-positive/HER-2 negative advanced breast cancer (Dhillon, 2015). The occurrence of infections in patients treated with palbociclib has been reported in several clinical trials. An open-label, randomized phase II study (PALOMA-1/TRIO-18) conducted in 2014 showed a higher incidence of infection-related AEs, including nasopharyngitis, stomatitis, influenza, and upper respiratory tract infection, in the palbociclib plus letrozole group than in the letrozole group (Finn et al., 2015). In 2016, a phase III, randomized PALOMA-2 study in postmenopausal women with ER-positive/HER2-negatvie advanced breast cancer further revealed that infection (any preferred term under the System Organ Class Infections and Infestations) was the most common serious AE among both Asian and non-Asian patients in the palbociclib plus letrozole group (Im et al., 2019). Consistent with the results of the PALOMA-1 and PALOMA-2 studies, the PALOMA-3 study in 2016 evaluating palbociclib in combination with fulvestrant for the treatment of women with HR-positive/HER2-negative advanced metastatic breast cancer showed that the palbociclib arm had a significantly higher incidence of infection-related AEs compared to the placebo arm (*p* < 0.02). Moreover, infection was the most frequently reported serious AE in the palbociclib group. Most infection-related AEs may be due to upper respiratory tract infections caused by viruses (Verma et al., 2016). Furthermore, xu et al. conducted a phase III PALOMA-4 trial in Asian postmenopausal women with ER-positive/HER2-negative advanced breast cancer in 2022 and found that the most common serious AEs in the palbociclib arm were infections (Xu et al., 2022). In the present study, the proportion of infection-related AEs associated with palbociclib was 2.72%. Case reports of serious outcomes for palbociclib accounted for approximately 30% of the cases. Most of the reports were from the United States. Based on the ROR method, fourteen infection-related PTs were strongly associated with palbociclib treatment.

The FDA granted accelerated approval for ribociclib in 2017 based mainly on the results of the MONALEESA-2 trial. The MONALEESA-2 study was a randomized, double-blind, placebo-controlled, phase III trial conducted in twenty-nine countries and showed that the incidence of infections in the ribociclib and placebo groups was 50.3% and 42.4%, respectively. Urinary tract infections and upper respiratory tract infections are the most common AEs but are generally grade 1 or 2 (Hortobagyi et al., 2016). In accordance with the results of the MONALEESA-2 study, Tripathy et al. conducted the MONALEESA-7 trial and found that the incidence of infection in the ribociclib and placebo groups was 47% and 37%, respectively. The most common AEs were urinary tract infections and upper respiratory tract infections, which were generally grade 1 or 2 (Tripathy et al., 2018). In addition, the MONALEESA-3 trial revealed that pneumonia (1.9% vs. 0%) was the most common all-grade all-causality serious AEs reported in no less than 1% of patients (ribociclib plus fulvestrant vs*.* placebo plus fulvestrant) (Slamon et al., 2018). Our study showed that infection-related AEs for ribociclib accounted for 8.58% in all AEs associated with ribociclib, which was the highest proportion of infection-related AEs among the three CDK4/6 inhibitors. Infection-related reports with serious outcomes for ribociclib accounted for 67.65% of cases. Most reports were from the United States and Germany. Sixteen infection-related PTs showed a strong relationship with ribociclib, according to the ROR method. Among these, the most frequently reported PTs was pneumonia; However, mastitis showed the strongest association with ribociclib treatment.

Abemaciclib, the third CDK4/6 inhibitor in the market, received FDA approval for the treatment of HR-positive/HER2-negative advanced or metastatic breast cancer in 2017 (Kim, 2017). The phase 3 MONARCH 2 study evaluating women with HR-positive/HER2-negative advanced breast cancer showed a higher incidence of infection in the abemaciclib arm (42.6%) than it did in the placebo arm (24.7%). The severity of these infections was generally grade 1 or 2, with grade 3 AEs accounting for only 6.6% of the abemaciclib arm and 3.6% of the placebo arm (Sledge et al., 2017). The phase 3 MONARCH 3 study showed that infections accounted for 39.1% of the patients in the abemaciclib arm and 28.6% in the placebo arm, of which grade 1 and 2 infections occurred in 33.3% of the abemaciclib arm and 25.5% of the placebo arm (Goetz et al., 2017). Furthermore, a subpopulation analysis in Japan from the MONARCH 3 study indicated that the incidence of pneumonitis of any grade was 10.5% in the abemaciclib arm and 6.7% in the placebo arm, while for grade ≥3, it was 2.6% in the abemaciclib arm and 0% in the placebo arm (Takahashi et al., 2022). In this study, infection-related AEs for abemaciclib accounted for 1.24% of all AEs associated with abemaciclib, and it presented the lowest number of infection-related AEs among the three CDK4/6 inhibitors. This is attributed to the distinct roles of CDKs. CDK4 is a prominent oncogene in breast cancer, and CDK6 is closely related to the differentiation of human hematopoietic stem cells. Abemaciclib shows higher selectivity for CDK4 and thus results in less myelosuppression compared to palbociclib or ribociclib (Braal et al., 2021). Infection-related reports with serious outcomes for abemaciclib accounted for 48.08% of the cases. Most of the reports were from the United States. According to the ROR method, two infection-related PTs showed a strong relationship with ribociclib.

This study had some limitations. This was a retrospective pharmacovigilance analysis based on data from the FAERS database. The FAERS database has some inherent biases, such as incomplete clinical characteristics of patients and infection types (Hauben et al., 2007). In addition, the incidence of AEs could not be evaluated because of the absence of a total population treated with these three CDK4/6 inhibitors. Furthermore, the disproportionality analysis only indicated a statistically significant association between these three CDK4/6 inhibitors and AEs but could not reveal whether there was a causal relationship. Therefore, these findings should be interpreted with caution. However, reports from the FAERS database provide a valuable assessment of post-marketing safety in real-world data to some extent.

## 5 Conclusion

Disproportionality analysis revealed that fourteen, sixteen and two infection-related PTs were detected for palbociclib, ribociclib, and abemaciclib, respectively. Ribociclib was not only more likely to cause infection, but also to cause serious outcomes compared to palbociclib and abemaciclib. However, the disproportionality analysis only indicated a statistically significant association, and further causality assessment is required.

## Data Availability

The original contributions presented in the study are included in the article/Supplementary Material, further inquiries can be directed to the corresponding authors.
